# Removal of Copper Ions from Wastewater: A Review

**DOI:** 10.3390/ijerph20053885

**Published:** 2023-02-22

**Authors:** Yongming Liu, Haishuang Wang, Yuanyuan Cui, Nan Chen

**Affiliations:** 1 Shandong Provincial Geo-Mineral Engineering Co., Ltd., Jinan 250013, China; 2 MOE Key Laboratory of Groundwater Circulation and Environmental Evolution, School of Water Resources and Environment, China University of Geosciences (Beijing), Beijing 100083, China; 3 Shandong Geological Exploration Institute of China Geology and Mine Bureau, Jinan 250013, China

**Keywords:** copper, wastewater, health, physical–chemical technology, biotechnology

## Abstract

Copper pollution of the world’s water resources is becoming increasingly serious and poses a serious threat to human health and aquatic ecosystems. With reported copper concentrations in wastewater ranging from approximately 2.5 mg/L to 10,000 mg/L, a summary of remediation techniques for different contamination scenarios is essential. Therefore, it is important to develop low-cost, feasible, and sustainable wastewater removal technologies. Various methods for the removal of heavy metals from wastewater have been extensively studied in recent years. This paper reviews the current methods used to treat Cu(II)-containing wastewater and evaluates these technologies and their health effects. These technologies include membrane separation, ion exchange, chemical precipitation, electrochemistry, adsorption, and biotechnology. Thus, in this paper, we review the efforts and technological advances made so far in the pursuit of more efficient removal and recovery of Cu(II) from industrial wastewater and compare the advantages and disadvantages of each technology in terms of research prospects, technical bottlenecks, and application scenarios. Meanwhile, this study points out that achieving low health risk effluent through technology coupling is the focus of future research.

## 1. Introduction

With the development of human life and industrial production, heavy metals pollution is becoming more and more serious and has become an environmental problem, which cannot be ignored [1]. Heavy metals mainly refer to elements with relative atomic masses between 63.5 and 200.6, specific gravity greater than 5.0, and atomic density greater than 4.5 g·cm^−3^ [2,3,4]. They are mostly transition metals and include more than 40 kinds of heavy metals, such as nickel, mercury, lead, copper, zinc, and cadmium. In response to the current study, heavy metals pollution in soil and water was found to be a widespread problem [1,5]. With the rapid economic development and population explosion, the water resources available for direct use on earth have been in shortage. Therefore, water pollution control has inevitably become a global concern [6,7]. In this study, the current status of heavy metal (Cu(II)) pollution, hazards, and treatment methods are reviewed with heavy metal polluted wastewater as the main entry point. As an important part of the earth’s ecosystem, water bodies are the most basic natural resources on which human beings depend, and heavy metals in air and soil can be released into water through atmospheric deposition, precipitation, and leaching [7,8]. Moreover, heavy metals are widely used in engineering, paper, fine chemical, dye, paint, pharmaceutical, petrochemical, and textile industries, which inevitably leads to excess concentration of heavy metals in wastewater [9,10]. Heavy metals are highly toxic elements, leading to the amplification of the entire food chain and adversely affecting human health and the environment [11,12]. Therefore, heavy metals pollution has received widespread attention.

Copper is a typical transition metal, which occurs widely in nature and is the third most used metal in the world, belonging to the group of heavy metals [12]. Copper, which is usually considered a highly hazardous heavy metal [13], is an essential element required by humans and plays a crucial role in enzyme synthesis, bone development, and tissues. The different forms of copper are Cu(0) (metal), Cu(I) (cuprous ion), and Cu(II) (copper ion), where Cu(II) is found to be the most toxic and occurring element in the environment. Cu(II) is widely used in electroplating, paints and dyes, petroleum refining, fertilizer, mining and metallurgy, explosives, pesticides, and steel industries and is considered one of the most important hazardous heavy metals in these industrial effluent streams [14]. In terms of human health, copper is an essential element for maintaining the vital activities and physical health of the human body, and it has an important impact on the development and physiological functions of the human blood and immune system, liver, heart, and eye organs. Copper deficiency can lead to anemia and defects in connective tissue, but too much copper can cause acute gastrointestinal symptoms, inactivation of enzyme systems in the liver, and even motor disorders in some patients with copper overdose [15,16,17]. In the aqueous environment, copper can permeate surface and groundwater systems and may also be transferred to drinking water, threatening human health. Cu(II) pollution has been on the rise in the global aqueous environment and has been identified as a major heavy metal contaminant due to the health risk (Table 1) [18,19]. There are many national and international reports on Cu(II) contamination in aqueous environments. Cu(II) concentrations measured in freshwater ecosystems in the United Kingdom ranged from 0.02 to 133 mg/L, and it was one of the heavy metals of greatest concern [19]. In the western part of the Netherlands, 39.1% of the area had excess Cu(II) concentrations in surface water [20]. In China, the Keelung area, Poyang Lake, as well as Wuhekou in Taiwan, were contaminated with heavy metals, and copper was the most prominent [21,22,23]. The ingestion, skin contact, and inhalation of copper will lead to health risk for local residents. Therefore, it is very important to understand the health risk probability for local residents living around copper smelters, the pollution level leading to health risks, and different treatment technologies. Therefore, the U.S. Environmental Protection Agency (EPA) and the World Health Organization (WHO) estimate that the maximum Cu(II) content in drinking water should be 1.3 and 3 mg/L, respectively [12]. Meanwhile, China stipulates in the Groundwater Environmental Quality Standards and Surface Water Environmental Quality Standards that the content of Cu(II) should be less than or equal to 1.5 and 1.0 mg/L (GB 3838-2002; GB 14848-2017).

Based on the above, the technologies regarding the treatment of Cu(II)-contaminated wastewater have been widely reviewed. The reported technologies for the removal of Cu(II) contamination mainly include physicochemical techniques as well as biological techniques, and surprising removal results have been obtained. Most of the existing review articles focus only on an in-depth description of a technique, extending to the mechanism of action, material selection, service life, and means of optimization. These articles are necessary for an in-depth investigation by researchers, but they are not conducive to a comprehensive understanding of the field by beginners. This review provides a comprehensive overview of the currently available copper treatment technologies and summarizes their potential advantages and disadvantages, which can help researchers select targeted water treatment methods. In particular, the advantages and disadvantages of various technical approaches differ greatly in terms of application scenarios, costs, and technologies. Taking into account the prevalence of Cu(II) contamination and the valence variability, which distinguishes it from arsenic, chromium, and vanadium, the biological removal techniques and mechanisms for Cu(II) are very different, and therefore, a summary of this research direction is also needed to provide ideas for subsequent researchers. In this paper, in addition to the presentation on physicochemical techniques, the applications of biotechnology in the treatment of Cu(II)-contaminated wastewater are included, which are hardly covered in previous reviews of similar type. Therefore, our current review focuses on the mechanistic differences, application adaptability, and technological advantages of different technological tools. This study provides theoretical support and technical guidance for basic research on Cu(II)-contaminated wastewater.

## 2. Restoration Techniques

**Figure 1:**
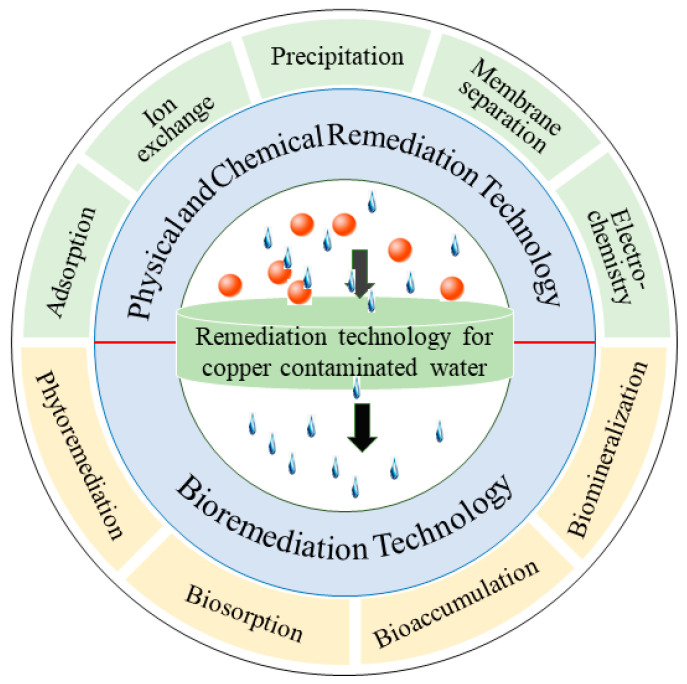
Treatment technology of Cu(II)-polluted wastewater.

So far, several technologies have been developed and utilized for Cu(II) removal from water and wastewater, and according to the reaction mechanism, the available studies classified these technologies as physicochemical (membrane separation, ion exchange, chemical precipitation, electrochemistry, adsorption) and biological (biosorption, bioprecipitation, biomineralization) [2,9,14,31] (Figure 1).

### 2.1. Physical–Chemical Technology

#### 2.1.1. Membrane Separation

Membrane separation methods mainly use the selective permeability of membranes to concentrate and separate heavy metals, and the common membrane separation technologies are microfiltration, ultrafiltration, nanofiltration, reverse osmosis, and electrodialysis [32].

##### Microfiltration, Ultrafiltration, and Nanofiltration

**Figure 2:**
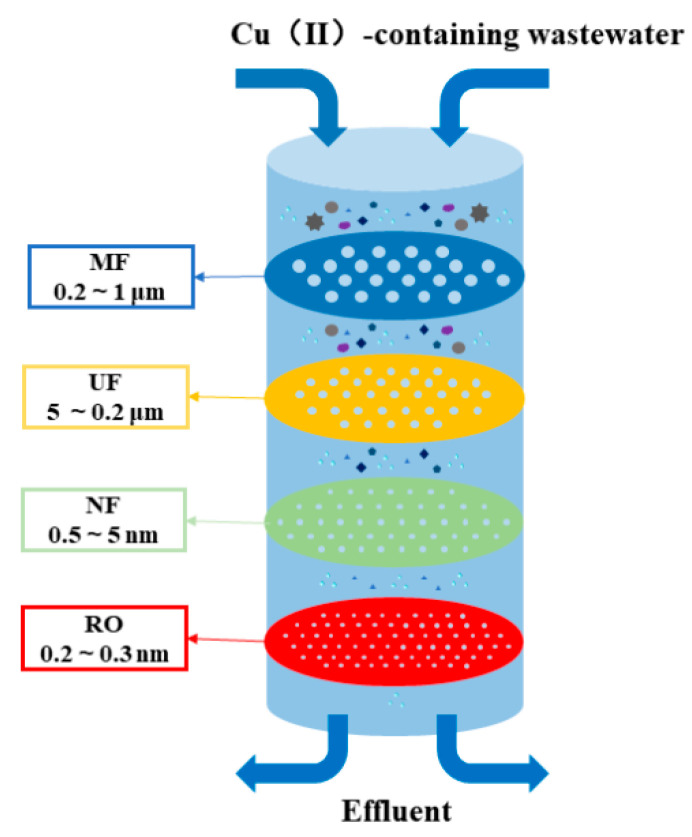
Membrane technology including MF, UF, NF, and RO.

The main difference between microfiltration, ultrafiltration, and nanofiltration is the pore size of the membrane: microfiltration (MF, 0.2–1 μm), ultrafiltration (UF, MW: 1000–1,000,000, 5–0.2 μm), nanofiltration (NF, MW: 100–1000, 0.5–5 nm), reverse osmosis (RO, MW < 100, 0.2–0.3 nm) [33] (Figure 2). Metal sulfide precipitation combined with microfiltration processes have been studied for Cu(II) recovery from acid mine drainage [34]. However, microfiltration was rarely used for heavy metals removal because its large pore size (100–1000 nm) was not an absolute barrier for heavy metals [3]. By preparing ultrafiltration hybrid matrix hollow fiber membranes impregnated with ZnO nanoparticles, the removal of Cu(II) exceeded 92% at a specific permeate flow rate of 0.115 L m^−2^h^−1^kPa^−1^, and the hybrid matrix membranes showed an adsorption capacity of 88 mg/g at pH 8. Ultrafiltration had a low ability to remove small molecular weight organics and was also limited in the removal of metal ions; therefore, polymer-enhanced ultrafiltration (PEUF) using polymer–metal interactions and membrane filtration was used for heavy metals removal. The removal of Cu(II) by PEUF up to 97% was investigated with polyvinylamine as a chelating agent [35]. Surfactants show great potential in removing metal contamination. However, it should be noted that the process of metal removal needs to be further improved, and the surfactant with the best metal removal effect should be selected from the perspective of environmental protection or metal recovery value. In addition, the utilization of recovered metals or metal solutions in the presence of surfactants should be studied from an electrochemical, ultrafiltration, or other technical point of view. At the same time, the method using surfactant mixtures is very advantageous for maximizing the treatment of multiple target metals. With low-pressure membrane separation processes (e.g., microfiltration and ultrafiltration), the removal of heavy metals was limited by their large pore size allowing the passage of heavy metal ions [36]. The current solution is to develop hybrid ultrafiltration/microfiltration processes or to combine membrane separation and electrochemical processes to prepare conductive membranes to achieve low-pressure membrane for metal ions removal [36]. Reverse osmosis technology has a high energy consumption and may remove ions from the body, which do not need to be removed [33].

Nanofiltration technology for the separation of Cu(II) from aqueous solutions has proven to be a viable technology for effective Cu(II) removal over a wide range of operations. Nanofiltration (NF) is used as a pressure-driven membrane technology located between ultrafiltration (UF) and reverse osmosis (RO). NF allows the passage of water molecules and most monovalent ions while rejecting most organic molecules, multivalent ions, and colloidal particles [37]. It has a lower working pressure than RO due to the loose selective layer and a better ion selectivity than UF due to the appropriate pore size [38]. This special separation capability has led to the wide application of nanofiltration membranes for hardness removal, heavy metal ion removal, and dye\salt separation. Nanofiltration membranes are usually composite products, and the substrates may include polyethersulfone (PES), polysulfone (PSF), polyvinylidene fluoride (PVDF), polyacrylonitrile (PAN), and polytetrafluoroethylene (PTFE), which provide the required mechanical strength to the membrane. In addition, the functional layer is critical to the filtration effectiveness of the composite membrane. The key factors include the thickness of the polymer layer, the pore size, and the selection of the support layer, which determines the membrane flux, selective permeability, and retention performance. To this end, the methods of preparing functional layers are important, including interfacial polymerization [39], cross-linked coatings [40], layer-by-layer self-assembly techniques [41], and surface hyperbranched modifications [33]. Qi et al. [42] prepared a novel positively charged nanofiltration membrane using 2-chloro-1-methylpyridine as the active agent and harvested more than 96% Cu(II) removal by covalently grafting polyimide polymers with surface carboxyl groups. Tian et al. [43] prepared a composite nanofiltration membrane to achieve an ideal rejection rate of Cu(II) exceeding 98%. Polymer-anchored co-deposition method consisting of positively charged hollow fiber nanofiltration membranes with a bridge network structure showed excellent removal efficiency for a high concentration of heavy metal ions (4000 mg/L) [44].

The spatial site resistance effect and Donnan exclusion are the two main separation mechanisms of NF [42,44]. Among them, the spatial site resistance effect, namely the sieve effect, is related to the molecular geometry (width). The Donnan effect refers to the repulsion of ions due to the surface charge of the nanofiltration membrane. Therefore, the surface charge of the NF membrane is modified to neutral or microcharge to enable the membrane to remove a large range of heavy metals, and adsorption mechanisms other than size exclusion and charge rejection are introduced to improve the rejection efficiency of the membrane [45]. Nanofiltration technology has good stability, low chemical consumption, energy efficiency, small carbon footprint, easy management and maintenance, and it can achieve zero emissions [46]. However, there are still many challenges in its application, such as membrane fouling, membrane pore size, and membrane material biodegradability. There is an emerging research trend to develop a natural/biodegradable polymer-based membrane with sustainable, high flux, and separation efficiency [33]. Fouling is a complex phenomenon resulting from the interaction between feed solution, membrane properties, and operating conditions [37]; therefore, membrane cleaning is essential to maintain membrane permeability and selectivity. This can be mitigated by electrolysis [47], ultrasonic cleaning [48], chemical cleaning [49], and backflushing [50]. Physical cleaning can alleviate membrane contamination and reduce the frequency of chemical cleaning, thus extending the membrane life and reducing operating costs. Although membrane fouling cannot be avoided, contamination can be reduced by adequate selection of membrane pore size and material and by controlling operating condition factors, such as transmembrane pressure, temperature, and flow rate [51].

Membrane separation processes have been identified as a viable option for the removal of heavy metals from aqueous solutions because they are easy to construct and control, and valuable metals can be recovered. However, high operating pressures, pH sensitivity, and the driving force of foreign ions limit their application. Therefore, understanding the separation behavior of a specific membrane process under various operating conditions is important to design a viable membrane process.

##### Reverse Osmosis and Electrodialysis

In reverse osmosis, the applied pressure difference is greater than the osmotic pressure difference across the membrane; therefore, water molecules are forced to flow in the opposite direction to the natural osmosis phenomenon [52]. Electrodialysis is an electro-membrane method, whereby ions are transferred by an electric current applied to the membrane [53]. Reverse osmosis and electrodialysis have continuous channels through which water and ions move. Membrane charge and chemical affinity cause solutes to be split on the two outer sides of the membrane. On the upstream side of the reverse osmosis membrane (reflux side), the ion concentration increases through the diffusion boundary layer toward the membrane (concentration polarization), and on the downstream side, a permeate is produced [54]. Reverse osmosis and electrodialysis also have three main aspects in common: the difference in concentration between the two sides of the membrane, the pressure difference across the membrane, and the presence of an electric current [54].

Reverse osmosis is the reverse process of osmosis and generally refers to the process of allowing the solvent to pass through a semi-permeable membrane and retain some or all of the solute under external pressure. There are two conditions to achieve reverse osmosis: first, the operating pressure must be greater than the osmotic pressure of the solution; second, there must be a highly selective, highly permeable semi-permeable membrane. In the treatment of heavy metals wastewater, the retention mechanism of reverse osmosis is mainly the sieving mechanism and electrostatic repulsion. Therefore, the retention effect of heavy metals is also related to the valence state of heavy metal ions [54,55]. Aromatic polyamide ultra-low-pressure reverse osmosis membranes have the ability to separate Cu(II), obtaining >95% metal rejection in synthetic and real industrial wastewaters [56]. Reverse osmosis membranes were combined with an electro-coupling process to achieve remediation of Cu(II)-contaminated water, and the effects of electrolysis voltage, pH, and electrolysis time on metal recovery efficiency, and the relationships between trans-membrane pressure drop (ΔP), addition rate, and initial Cu concentration and operating efficiency, membrane stability, and water reuse potential, were investigated [57]. Numerous scientific experiments have demonstrated the excellent effectiveness of reverse osmosis membranes for Cu(II) removal, especially for high-Cu(II) contamination loads and contaminant ions’ coexistence [58,59]. Pilot-scale membrane bioreactor systems in combination with reverse osmosis had a very high heavy metals removal efficiency [59]. In addition, the combination of reverse osmosis and nanofiltration for efficient heavy metals removal was well reported. The combination of nanofiltration and reverse osmosis membranes was effective in removing Cu(II) from the water of a textile coating plant [60]. However, the RO process is also subject to membrane fouling and blockage problems, and vibrational shear-enhanced treatment techniques combined with conventional RO membranes extract valuable heavy metals from concentrates [61]. The main drawback of reverse osmosis is the high power consumption due to pump pressure and membrane repair, which is the focus of future research.

In electrodialysis, the water flows through thin channels next to an ion exchange membrane, and the applied current pulls ions from one set of channels through the IEM to other channels [54]. Electrodialysis is an electrically driven separation process, which can be easily scaled up and used in combination with other processes [62]. Electrodialysis has proven to be very effective in the removal of Cu and Fe from working solutions [63]. Ion exchange membranes, which are the core of ED systems, are semi-permeable to ions due to the fixed ionic groups on their backbone [62]. The preparation of ion-exchange membranes with desirable permeability, low resistance, improved thermal, chemical, and mechanical properties, and high cost effectiveness is the focus of research in this technology. In addition, electrodialysis exhibits high selectivity in separations and high energy efficiency at high operating costs.

#### 2.1.2. Ion Exchange

The ion exchange process has been successfully used to remove heavy metals from industrial wastewater, especially from acidic wastewater. The ion exchange uses the free ions carried by the solid phase exchanger itself to exchange with the heavy metal ions in the liquid phase to separate the metal ions from the wastewater. Exchange resin is a common exchange agent, and the exchange resins obtained by different preparation methods have different affinities for metal ions, that is, they have selectivity for different metal ions [64]. Among the materials used in ion exchange, synthetic resins are usually preferred because they are almost effective in removing heavy metals [65]. Adsorption selectivity and capacity are the two most important properties of resins. The selectivity of adsorption comes mainly from the interaction between the adsorbent and the functional groups on the surface of the chelating resin, so the type of functional group plays a crucial role. The functional groups on the surface of chelate resin not only affect the adsorption selectivity, but they also dominate the adsorption mechanism. The tert-butyl 2-methylamino-N-acetic acid functionalized chelating resin could remove trace copper from simulated nickel electrolytes with high selectivity [66]. Magnetic cation exchange resin synergistically removed Cu(II) and tetracycline (TC) from their mixed solutions and had great potential for application with negligible loss of adsorption capacity over five adsorption–desorption cycles [67]. The commercial resin MTS9600^®^ containing dichloramine groups was used to selectively separate nickel and copper from acidic effluents of sulfate media with 99% copper removal at selected operating conditions (pH = 2.0) [68]. Ion-exchange technology has been successfully applied in the recovery of hydrometallurgical lithium-ion battery waste, and aminomethylphosphonic acid functional group chelating resin (Lewatit TP260) was able to remove Fe, Al, Mn, and Cu from the leachate [69]. In fact, Murray and Örmeci have developed nano- or submicron-sized adsorbents as alternatives to conventional adsorbents, which were able to remove 46% ± 0.6% of copper from river water spiked with 500 μg/L and 38% ± 0.8% of copper from actual wastewater [70]. Moreover, in addition to synthetic resins, natural zeolites were widely used for the removal of heavy metals from aqueous solutions due to their low cost and high abundance. Additionally, it has been shown that zeolites exhibit good cation exchange capacity for heavy metal ions under different experimental conditions.

The main advantages of ion-exchange technology are high uptake of the target material, fast reaction kinetics, efficient elution, and lifetime durability [71]. However, the feasibility of the ion-exchange resin process depends heavily on the long-term reusability of the resin and the possibility of recovering the target compound from the regenerated solution. Typically, adsorbed Cu(II) was released by washing with concentrated acid (1.0–2.0 M H_2_SO_4_), which protonated the nitrogen sites. To improve the recovery of Cu(II), additional washing with concentrated aqueous ammonia solution (1.0–2.0 M NH_4_OH) is required to completely release the metal [72]. The design of an efficient chelating resin elution scheme needs to be refined in subsequent experiments to achieve efficient heavy metal recovery.

#### 2.1.3. Electrochemistry

Electrochemical technologies are used to achieve the desired purpose through a series of chemical reactions, electrochemical or physical processes. They have some special advantages compared to traditional wastewater treatment methods. (1) Electrochemical technologies are versatile and can be used not only for the degradation and transformation of pollutants but also for suspension systems or colloidal systems. They can play a role in the treatment of wastewater, exhaust gases, and toxic waste. The main parameters of the electrochemical process are potential and current, which are easy to measure and control. (2) The electrochemical reaction process does not require the addition of chemicals to avoid secondary contamination. (3) Electrochemical treatment equipment is relatively simple, with high removal efficiency and low operation and maintenance costs. (4) The amount of sludge (the precipitates produced during flocculation or deposition) produced is small, the post-processing process is simple, and the operating area is small. According to different electrode materials and electrode reactions, electrochemical methods can be mainly divided into electrodeposition and electroflocculation.

##### Electrodeposition

Electrodeposition can recover metal ions by selective removal and can even be used to produce new materials, and it is widely used in heavy metal wastewater treatment [73]. Carpanedo de Morais Nepel et al. [74] studied and optimized the recovery of copper from real wastewater by pulsed electrodeposition, using fast current pulses (ton = 1 ms, 190 mA, 70 rpm, 37 °C) in an experiment with a deposition efficiency of 84.36% and a copper removal of 33.59% in 30 min, obtaining 100% purity of copper metal and crystalline copper in the coating. However, electrodeposition has the disadvantages of low treatment efficiency, long treatment time, and high energy consumption in the treatment of heavy metal wastewater containing Cu(II) due to the reduction potential and mass transfer process of metal electrodeposition, which limit its application.

##### Electroflocculation

Electroflocculation was able to generate a large number of cations at the anode of the external power supply to generate a series of polynuclear hydroxyl complexes and hydroxyl ions, from which suspended solids and organics were adsorbed. At the same time, the cathode generated hydrogen, which gathered into micro foam and rose to the surface to form a contact suspension layer, thus purifying the wastewater. Wu et al. [75] used DC electrocoagulation flocculation to treat alkali–ammonia corrosion wastewater from printed circuit boards with an electrode distance of 28 mm and a current density of 100–300 A·m^−2^, which could effectively remove Cu(II) from alkali–ammonia corrosion wastewater, with the recovery of Cu(II) exceeding 99%. Electroflocculation has many advantages, including simplicity of operation, high removal efficiency, and low sludge (the precipitates produced during flocculation) formation rate [76,77]. However, a major drawback of electrochemical flocculation is that it requires a large amount of electricity proportional to the initial concentration of heavy metals [78]. Therefore, reducing the heavy metal concentration prior to electrochemical treatment will reduce the overall electricity demand. Mohammad Rahimi et al. [37] modified a thermally regenerated ammonia battery (TRAB) using waste heat and power generation and used it as a treatment process for solutions containing high concentrations of Cu(II), showing that the initial Cu(II) concentration of 0.05 mol/L resulted in a high copper removal rate of 77% and a maximum power density of 31 W·m^−2^. The modified TRAB was a promising technology for the removal of Cu(II) as well as for the use of waste heat as a high-availability and free energy source for power generation in many industrial sites.

#### 2.1.4. Chemical Precipitation

Dissolved metal ions are converted to an insoluble solid phase by chemical reaction with a precipitant (e.g., base or sulfide), and the resulting precipitate can be separated from the water by precipitation or filtration. Traditional chemical precipitation processes mainly include hydroxide precipitation and sulfide precipitation. Chemical precipitation is most widely used in industry, mainly because the simplicity of process control allows it to be effective over a wide range of temperatures and at low operating costs [79]. Inorganic precipitants commonly used for heavy metal precipitation are lime (Ca(OH)_2_), caustic soda (NaOH), soda ash (Na_2_CO_3_), sodium bicarbonate (Na(HCO_3_)_2_), sodium sulfide (Na_2_S), and sodium hydride (NaHS) [80,81]. Chemical precipitation uses pH adjustment to convert heavy metal ions into hydroxides, sulfides, carbonates, or other less soluble compounds, which are then removed by physical means (e.g., precipitation, flotation, or filtration) [76]. Chemical precipitation has the advantages of low cost, simplicity of operation, as well as non-metallic selectivity. Notably, the chemical precipitation method often introduces a large number of inorganic ions into the wastewater, leading to high salinity when removing Cu(II) due to the need to add additional agents or adjust the pH value, leading to an extreme (acid/base) pH environment, which makes it difficult to achieve environmentally friendly effluent quality. This makes chemical precipitation more suitable for high concentrations of Cu(II) wastewater, such as acidic mine wastewater.

##### Hydroxide Precipitation

The precipitation of soluble metals into insoluble hydroxide form with lime in an alkaline environment was proposed as early as the 1880s [77]. Currently, neutralization precipitation of inexpensive CaO is the most widely used process in the treatment of waste acid wastewater from copper smelting because of its low cost and simplicity of operation [82,83]. In pilot-scale experiments, the optimal pH for achieving maximum copper precipitation with lime and caustic soda used in the hydroxide precipitation method was determined to be around 12.0 [80]. Wang et al. used the bicarbonate-activated hydrogen peroxide/chemical precipitation method to simultaneously perform Cu-EDTA depolymerization and Cu(II) precipitation. It was found that the composition of the precipitate was identified as CuCO_3_, Cu_2_(OH)_2_CO_3_, Cu(OH)_2_, CuO, and/or CuO_2_, and TOC removal efficiency and Cu removal efficiency reached 78.4% and 68.3% after 60 min treatment, respectively [81].

##### Sulfide Precipitation

Metal sulfide species are highly insoluble, especially for copper with logK_sp_ values between −49.2 and −35.9 [84]. This fact is an attractive advantage for environmental applications, especially in terms of chemical stability. By comparison, metal sulfide precipitation is superior to metal hydroxide precipitation because of (1) the high reactivity of sulfides with heavy metal ions and the very low solubility of metal sulfides over a wide pH range. (2) The metal sulfide sludge (the precipitates produced during chemical reactions) is denser and has better thickening and dewatering properties than metal hydroxide sludge; and (3) metal sulfides are good selective precipitators and are insensitive to the presence of complexes [33].

Other chemical precipitation methods, such as the classical alkaline precipitation method, form difficult-to-eliminate heavy metal complexes due to the strong bonding ability between Cu(II) and EDTA [85]. However, the process of metal separation and recovery during chemical precipitation still needs to be further addressed [83]. For example, the recovery of Cu(II)–EDTA in Cu–organic-compound contaminated wastewater was difficult due to its high stability, resulting in a “replacement–precipitation” strategy, whereby the affinities of the replacement agent (stronger Ca replacement agents (Ca and Fe)) were investigated [85].

#### 2.1.5. Adsorption

**Figure 3:**
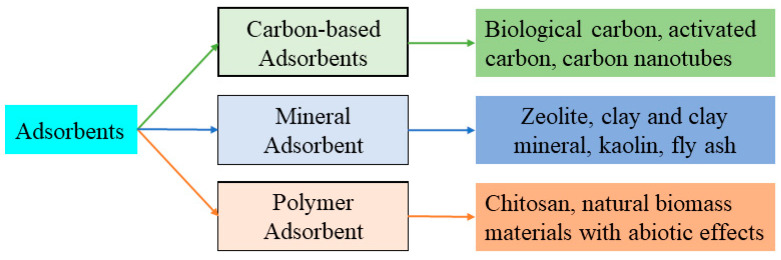
Common adsorbent types in Cu(II) removal process.

Adsorption methods include physisorption and chemisorption, where physisorption is the adsorption of an adsorbent by van der Waals forces; chemisorption is the adsorption of an adsorbent by chemical bonding; and biosorption is adsorption by proteins secreted by organisms (bacteria, fungi, and algae). In general, the Gibbs free energy of physical adsorption (physisorption) varies between −20 and 0 kJ/mol; however, chemisorption ranges from −400 to −80 kJ/mol [86]. During the adsorption process, both adsorption pathways can exist separately, occur simultaneously, or be dominated by one or the other. Usually, we do not make a clear distinction between physical and chemical adsorption and collectively refer to them as adsorption. Adsorption is a method for adsorbing heavy metal ions using the well-developed pore structure, high specific surface area, and abundant functional groups on the adsorbent surface, which is an efficient, operable, and economical method for aqueous phase Cu(II) remediation [87]. In the adsorption process, the selection of an adsorbent with excellent adsorption efficiency is key to the adsorption technique. Many researchers have used various adsorbents, such as activated carbon, zeolite, activated alumina, lignite coke, bentonite, ash, clay, and natural fibers, to remove heavy metal ions from aqueous solutions. Adsorption efficiency and selectivity mainly depend on the chemical and physical properties of the adsorbent [88]. The common types of adsorbents can be classified according to the type of material as carbon-based adsorbents, natural mineral adsorbents, and natural polymer adsorbents (Figure 3).

##### Carbon-Based Adsorbents

Biochar is a carbon-rich solid obtained by pyrolysis of biological waste under low-temperature and limited oxygen conditions [89]. With a high specific surface area, well-developed porous structure, and high thermal stability, biochar shows great potential for immobilization of heavy metals. Zhou et al. [90] found that the main adsorption mechanism of Cu(II) with biochar of tobacco stems was related to surface complexation. Chen et al. [91] showed that the maximum adsorption capacity of corn stover biochar for Cu(II) was 12.5 mg/g. The adsorption capacity of Cu(II) was 71.4 mg/g in lobster shell-based biochar via cation exchange, mineral precipitation, and interactions such as functional group complexation and π-electron coordination with biochar [92]. The disadvantages of raw biochar, such as surface hydrophobicity, low number of functional groups, and weak metal binding ability, limit its ability to purify heavy metals wastewater [93]. Therefore, the development of green, simple, and economical modification methods to improve its adsorption capacity for heavy metal ions has become a priority. Biochar can be activated physically or chemically, depending on the desired surface properties, and the activation usually includes physical activation (steam or carbon dioxide) as well as chemical activation (zinc chloride, phosphoric acid, potassium hydroxide, and sodium hydroxide) [94,95,96]. Activated carbon is a black solid substance similar to granular or powdered charcoal, a carbonaceous material with highly developed porosity, high specific surface area, and relatively high mechanical strength [97]. Amorphous MnO-embedded porous rubber seed shell biochar prepared by KMnO_4_ impregnation–coking activation treatment efficiently purified Cu(II)-containing wastewater in a wide pH range (>2) and increased the equilibrium adsorption capacity of Cu(II) by 3.88 times (200.59 mg/g) [93]. The modification of larch biochar with wood ash as a modifier increased the maximum removal of Cu(II) by 9.66–11.11 times (38.9 ± 2.4 mg/g, 33.8 ± 2.3 mg/g), as the alkaline cations in wood ash increased the cation exchange process occurring on the biochar surface [98]. Biochar modification enhanced the intrinsic properties, such as surface area, porosity, morphology, and functional groups. The methods of biochar modification include metal impregnation, magnetization, and activation [99]. Activated carbon adsorption was widely used due to its porous surface structure and was environmentally benign and easy to handle [100]. However, the high cost of activated carbon limited its application, and therefore, there is a need to find alternatives to investigate low-cost, effective, and economical adsorbents. Waste rubber tires and a wide variety of agricultural wastes, such as orange peel, banana peel, peat, wood, pine bark, soybean and cotton seed shells, shells, hazelnut shells, peanuts, rice husks, wool, sawdust, compost, and leaves, have been made into activated carbon adsorbents [101]. In addition, carbon nanotubes were considered an effective heavy metal adsorbent because of their stability, large specific surface area, good mechanical properties, and high adsorption capacity [102].

##### Mineral Adsorbents

Zeolite is a porous aluminosilicate crystal with a tetrahedral structure based on TO_4_ (T = Si or Al). Zeolites are widely used in the removal of heavy metals from water due to their high affinity for specific contaminants [95]. Low-value materials are used to prepare zeolites to reduce the environmental impact and cut costs, such as fly ash [96], kaolin [103], red mud [104], and lithium silica powder [95]. Among other things, this enables the resourceization of waste while adsorbing and recovering heavy metals, which has a win-win effect. Furthermore, clay as an adsorbent has many advantages over other commercially available adsorbents in terms of low cost, abundant availability, high specific surface area, excellent adsorption properties, non-toxic nature, and ion-exchange potential [105]. Clays and clay minerals (montmorillonite, kaolinite, and illite) have a small particle size and complex porous structure with high specific surface area, which allows strong physical and chemical interactions with dissolved substances. These interactions are due to electrostatic repulsion, crystallinity, and adsorption or specific cation exchange [105]. Most clay minerals are negatively charged and very effective, and they are widely used to adsorb metal cations from solutions due to their high cation exchange capacity, high surface area, and pore volume. The absorption of heavy metals by clay minerals involves a series of complex adsorption mechanisms, such as direct binding of metal cations with the surface of clay minerals, surface complexation, and ion exchange [105]. Kaolinite obtained from Longyan, China, has good adsorption of Cu(II) under various conditions (metal ion concentration, clay amount, pH). It reaches maximum adsorption rapidly, within 30 min for Cu(II) [106]. However, the adsorption capacity of natural materials is low and needs to be modified to improve the separation efficiency and selectivity [107].

##### Polymer Adsorbents

Polymer adsorbent has a variety of functional groups on its surface, and these groups can combine with heavy metal ions in water to achieve the removal of metal ions from water. Natural polymer adsorbent mainly refers to chitosan, starch, lignin, cellulose, and other natural macromolecular substances with adsorption capacity. Chitosan is the second largest natural macromolecular compound besides cellulose [12], mainly found in insect shells, shrimp shells, crab shells, or cell walls of some micro-organisms, and the abundant amino and hydroxyl groups on its surface can be used to chelate heavy metal ions. Benavente et al. [108] prepared chitosan materials from shrimp shell waste with a maximum adsorption capacity of 79.94 mg/g of Cu(II). The limited functionality, solubility in acidic media, poor mechanical properties, and high swelling rate of typical chitosan-based adsorbents limit their applications, which can be functionalized by chemical oxidation, esterification, lipidation, and diazotization of chitosan backbone [13,109].

### 2.2. Biotechnology

**Figure 4:**
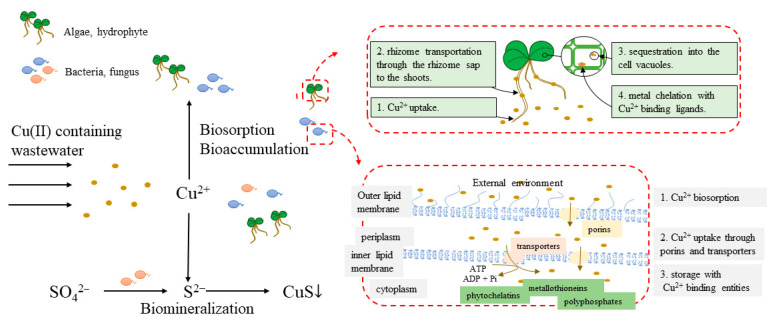
Biotechnology for copper removal.

The biological removal process of Cu(II) in the water environment mainly includes biosorption, bioaccumulation and biomineralization, and phytoremediation. The removal of Cu(II) by micro-organisms can be divided into two processes. One process involves the resistance gene of micro-organisms, which enables micro-organisms to survive and grow in the presence of Cu(II), and at the same time, Cu(II) can be accumulated in cells through cell membranes. The other process involves Cu(II), which can be adsorbed to organisms through physical and chemical actions by secreting EPS and other substances with adsorption capacity (Figure 4).

Biosorption, that is, heavy metals removal using cheap biological materials, such as algae, fungi, and bacteria, is becoming a potential alternative method for removing toxic metals from water [110]. One of the main advantages of biosorbents is that they are non-toxic and safe for the environment. Biosorption of heavy metals by metabolically inactive abiotic biomass of microbial or plant origin was an innovative and alternative technology for the removal of heavy metals from aqueous solutions [111]. Due to its unique chemical composition, biomass sequestered metal ions by forming metal complexes from the solution. The main mechanism involving the biosorption of metals (Pb^2+^, Ni^2+^, Cd^2+^, Cu^2+^, and Zn^2+^) using dead, dry aquatic plants as simple biosorbent materials for metal removal was the ion exchange between monovalent metals present in macrophyte biomass as counter ions and heavy metal ions and protons absorbed from water [110]. Seaweeds have a high binding affinity for heavy metals, and their cell walls have different functional groups (e.g., carboxyl, hydroxyl, phosphate, or amine), which can bind metal ions [112]. The seaweed *U. lactuca* from the Mediterranean coast of Egypt had a high polymetallic biosorption capacity, with a maximum biosorption efficiency of 64.51 mg/g for Cu(II) [113]. Compared with the physical and chemical methods, bacterial biosorption is a milder treatment method for toxic pollutants, which are not easily removed, such as heavy metals. These metal-tolerant bacteria can bind cationic toxic heavy metals to negatively charged bacterial structures and live or dead biomass components. Moreover, these bacterial biomasses can effectively act as biosorbents for metal bioremediation under polymetallic conditions due to the large surface area to volume ratio [114]. The biosorption process is based on the properties of microbial cell walls, consisting of different polysaccharides, proteins, and lipids, which provide a variety of functional groups (carboxyl, hydroxyl, phosphate, amino, sulfur) that can interact chemically with pollutants in a variety of ways [115]. For example, Cu^2+^ can react with these functional groups and result in organic metal precipitates [115]. These precipitates are removed from the bulk solution by adsorption on microbial cells. Similar metal cations, such as Ni(II), Cd(II), Cr(III), Cr(VI), and Co(II), can usually be removed by *Escherichia coli* C90, which is a commonly used method [110]. Microbial-based biosorption has several advantages in the removal of metal ions because it is selective for specific metals. In addition, the small size of micro-organisms provides a large specific surface area and volume for heavy metal adsorption. Additionally, due to the reusable nature of the biosorbents, the method is economically feasible and leaves minimal waste.

Biosorption is a metabolism-dependent mechanism, which enables the adsorption of contaminants onto cellular polymers [116]. Biosorption involves several mechanisms, including ion exchange, surface complexation, and physical adsorption. In general, ion exchange plays an important role in metal biosorption due to the electrostatic interactions that occur between the positive charge of free metal ions and the negative charge of the microbial cell wall [117]. In bacteria, the reactivity of the cell wall toward metals is mainly due to the presence of proactive functional groups, such as carboxyl, phosphoryl, hydroxyl, amino, and sulfhydryl groups, which can immobilize cations when deprotonated [118]. Unlike bioaccumulation, biosorption is rapid and reversible [119], but its efficiency depends on a variety of environmental conditions, especially the pH, which determines the charge of the microbial cell wall, but also on the ionic strength, the level of dissolved organic matter, and the metal concentration. Some authors claimed that the ion-exchange mechanism on the cell surface may be related to the metal removal mechanism in aqueous solutions. Electronegative elements may be responsible for metal biosorption [120]. Moreover, metals can also be adsorbed by extracellular polymers secreted by most environmental bacteria, which have a high affinity for copper. In addition to adsorption, some micro-organisms also removed copper by intracellular chelation precipitation, which reduced interference with cellular activity and enzymatic denaturation [121].

Bioaccumulation is an accumulation of contaminants regulated by microbial metabolic activity [119], which occurs when the rate of contaminant adsorption by micro-organisms is higher than the rate of contaminant loss through excretion. Among others, CPx-type ATPases played a role in the copper uptake capacity of some strains [122]. In turn, the bioaccumulation efficiency depended on the concentration of contaminants accumulated by the micro-organism. For example, *Amycolatosis tucumanensis* was able to accumulate up to 25 mg/g (dry weight) of copper, 60% of which was intracellular [123], and this particular bacterial species was able to efficiently trap copper within the cytoplasm by binding low molecular weight cysteine-rich proteins (metallothioneins) [124]. Depending on the location of metal uptake/accumulation, biosorption can be divided into extracellular precipitation, cell surface adsorption, and intracellular accumulation. In metabolism-dependent biosorption, living cell systems underwent biosorption and accumulated intracellularly [125]. Moreover, *Kluveromyces marxianus*, *Candida* spp., and *Saccharomyces cerevisiae* could remove 73–90% of copper during growth [126]. Another way was metal uptake by metabolism-independent biosorption, which occurs through physicochemical interactions between functional groups on the bacterial surface and metal ions. The binding of metal ions to bacterial cell surfaces in metabolism-independent biosorption involves various mechanisms, such as physical interactions (electrostatic or van der Waals interactions), chemical interactions (replacement of attached metal cations by ion exchange), complexation, diffusion, surface adsorption, or precipitation [127,128]. Bacteria reacted to harsh environments, such as heavy metal contamination sites, by releasing extracellular polymers (EPS) from the cell surface, which have a high affinity for copper [128]. In addition, proteins capable of chelating metal ions were detected in the supernatant of cells exposed to Cu(II) according to CELLO v2.5 [129].

The biomineralization mechanism relies on the ability of micro-organisms to create local supersaturation conditions, where metals are precipitated in solution by coming into direct contact with bacterial cells or their extracellular compounds. Thus, metals can be directly precipitated with anions released by micro-organisms, such as phosphates, which are less soluble for metals, or by replacing suitable cations from the lattice. In addition, micro-organisms can indirectly contribute to the “immobilization” of metals by influencing certain physicochemical parameters, which control the “solubility” of metals. For example, sulfate-reducing bacteria precipitate metals in the form of insoluble sulfides.

Long-term exposure to metal contamination results in microbial communities adapted to survive and persist in contaminated environments. In this sense, micro-organisms have developed complex and specific cellular mechanisms composed of a wide network of specialized proteins, transport proteins, and proteins involved in the regulation of gene expression in response to both metal deficiency and excess [130]. These cellular mechanisms, which maintain the optimal concentrations of metals, are called homeostasis. Micro-organisms play an important role in the uptake of metals from the environment by using a variety of mechanisms. These mechanisms differ between genera and/or microbial species, and little is known about them at the molecular level when in equilibrium condition [129].

Micro-organisms can mediate the immobilization of copper through biosorption, bioaccumulation, and biomineralization, as well as its activation through redox, acidolysis, or complexation decomposition of copper-containing phases [118]. Therefore, biological methods have advantages in the adsorption and release of copper. Microbial remediation of heavy metals has been used in the removal of heavy metal contamination due to its outstanding advantages of high efficiency and low cost. However, there are still many bottlenecks in its wide application. The molecular mechanisms of heavy metals detoxification need to be further elucidated to enhance the accumulation of heavy metal ions by micro-organisms. Extracellular/intracellular sequestration, active export, and enzymatic detoxification are the main resistance mechanisms of living micro-organisms to heavy metal ions, which would reduce their toxicity and convert them to inactive forms. Hydrogen sulfide precipitated heavy metal ions, and reductase altered the redox state of heavy metal ions, improving microbial resistance to heavy metal ions while achieving heavy metal remediation. There is a close internal inter-relationship between microbial resistance mechanisms to heavy metal ions and their repair capacity [131].

Plants are also able to tolerate and even resist copper toxicity under different environmental conditions. These include the release of organic acids into the soil to reduce copper bioavailability [132], complexation with cytosolic ligands to detoxify intracellular copper, and sequestering copper in intracellular compartments (e.g., vesicles) where the metal is least harmful. The tolerance of plants to copper is different among species and different varieties of the same species. In addition, severe copper phytotoxicity symptoms were observed in some copper-contaminated sites [133], and bacteria can enhance the tolerance of some plants to copper toxicity and can be used for revegetation in these areas.

## 3. Copper-Containing AMD Treatment Technology

Mining and processing plant flotation technologies also generate large amounts of acid mine wastewater (AMD) containing Cu(II), and these water characteristics distinguish them from conventional Cu(II)-containing wastewater, which not only contains large amounts of heavy metals (Cu(II)) but also has a low pH and is difficult to treat. The current treatment options for AMD are classified as passive or active processes [134]. The addition of various acid neutralization and metal precipitation chemicals (caustic soda (sodium hydroxide), lime and limestone, magnesium oxide, and hydroxide) to AMD water is a common active treatment method, which can meet wastewater discharge limits in a short period of time. The choice of chemical reagents depends on site specificity (seasonal variation), AMD influent loading, and metal concentration [135]. The advantages of the method are that it is fast and does not require additional operating sites. However, active treatment is usually considered expensive compared to passive treatment, and there are problems with disposal of aqueous sludge containing heavy metals. This method is mainly used for “active remediation” of short-term contamination.

Passive treatment is based on the advantages of naturally occurring geochemical and biological processes to improve the quality of AMD with minimal operational and maintenance requirements [136]. Moreover, passive treatment includes artificial wetlands, anaerobic sulfate reduction bioreactors, anoxic limestone drains, open limestone channels, limestone leaching beds, and slag leaching beds [135]. Passive systems can provide long-term, efficient, and effective treatment for many acid mine drainage (AMD) sources, provided they are properly planned and constructed, and require regular inspection and maintenance [137]. Most passive treatment systems employ multiple methods, often in series, to promote acid neutralization, oxidation, and precipitation of the resulting metal flocs. The conditions and chemistry of AMD, flow rates, acidity and alkalinity, metals, and dissolved oxygen concentrations are key parameters, which must be characterized before selecting the appropriate treatment technique. Passive treatment results in long cycle times and slow results but low environmental risk and low cost for long-term treatment of contaminated sites.

## 4. Conclusions and Outlooks for Cu(II) Removal and Recovery

Among the various pollutants, Cu(II) is one of the harmful heavy metals. It is discharged daily into wastewater streams from various industries, such as electroplating, paints and dyes, petroleum refining, fertilizers, mining and metallurgy, explosives, pesticides, and steel. Epidemiological studies have found an association between copper mining activities and various health diseases (e.g., headaches, cirrhosis, kidney failure, and even cancer) in people living near copper mining areas [138], and copper presents a high risk of cancer. Both maximum contaminant level goal (MCLG) and maximum contaminant level (MCL) for copper are 1.3 mg/L, meaning that there is no known or expected risk to health in drinking water below this level (https://www.epa.gov/, accessed on 9 October 2022). Potential ecological risk factors (PERF) and potential ecological risk index (PERI) are commonly used to assess environmental risk [139]. PERF ≤ 40 and PERI ≤ 150 are defined as low for both single and environmental risks.

Despite presenting a large environmental risk in water, Cu(II) is a critical metal to many industries, and removing Cu(II) from wastewater and considering the feasibility of Cu(II) recovery are promising strategies. This review explores the recent advances in Cu(II) removal technologies in water and wastewater. Although all heavy metal wastewater treatment technologies can be used to remove heavy metals, they have their inherent advantages and limitations in Cu(II) removal and even the separation and recovery prospects of Cu(II).

(i) Membrane separation is the most widely used technology for Cu(II) treatment in industry, which is able to concentrate and purify heavy metals while removing contaminants for later recovery. However, membrane fouling is always an obstacle limiting its process efficiency, so there is a need to develop cost-effective, efficient, and environmentally friendly flushing technologies.

(ii) The ion-exchange method has a high contaminant removal capacity, fast removal rate, efficient elution, and lifetime durability. The efficient elution is beneficial for Cu(II) recovery, but the long-term reusability of the resin and the possibility of recovering the target compounds from the regenerated solution limit the application.

(iii) Electrochemical technology has the advantages of simplicity of operation, high removal efficiency, and low sludge (the precipitates produced during flocculation or deposition) formation, but the high cost of electricity and separation increases the cost of its application.

(iv) Chemical precipitation is cost effective, simple, and non-metallically selective, but the high stability of the precipitate makes recovery difficult, resulting in a “replacement–precipitation” strategy, which requires research into more affinity-based replacement agents.

(v) The adsorption method has the advantages of simple operation, low cost, easy availability of materials, fast reaction rate, and good treatment effect, but in practice, the general adsorption materials may have low adsorption capacity, poor stability, and difficult separation after adsorption and need to improve the performance through physical or chemical modification.

(vi) The bioremediation method of removing Cu(II) from wastewater by algae, fungi, and plants is environmentally friendly and has little secondary pollution. However, the physicochemical properties of water can affect the performance of biosorbents. Biomineralization precipitation also seems to be effective in the removal of Cu(II), but the problem with this technique is the generation of metal-rich sludge (bioactive sludge), which makes the recovery of precipitated metals difficult. In addition, biological methods have high additional costs, such as the need for nutrients and regulation of the environment (pH, temperature), to maintain the biological process.

By combining the advantages and disadvantages of different technological approaches, coupling between technologies to achieve efficient copper removal and recovery as well as to obtain low health risk effluent is the focus of future research (Table 2 and Table 3). The review of technologies shows that conventional heavy metal treatment technologies are universal and can be useful in the removal of many heavy metals. However, different functional groups or selective resins have advantages in the removal of Cu(II), so we can optimize the material or technical means for this purpose. Meanwhile, the future research should focus on reducing system costs, improving efficiency, and developing intelligent systems. All technologies have their merits, and their use depends on their feasibility. Most studies have reported batch and laboratory-scale systems. Therefore, continuous systems and pilot-scale studies are needed to demonstrate industrial applications. In addition, real wastewater should be studied more than synthetic wastewater to investigate the real interaction of the technology with compounds in solution. Likewise, research should focus on commercialized technologies in the area of pollutant removal, so that the next generation of wastewater treatment can be developed in a sustainable, efficient, and cost-effective manner. Health risk assessment is also an important issue in the pollution management process. Considering the high toxicity response factor of copper (TR = 5) for different water bodies, we have to obtain an effluent with discharge concentrations lower than MCL to effectively avoid possible health risks.

## Figures and Tables

**Table 1:** Cu concentrations around the world.

Country/Region	Concentration	Paper
China/Tibet, Rona	Water: 2114.00 ± 65.89 μg/L Soil: 19.01–1763.1 mg/kg	[23]
China/Yunnan Copper Mine WWTP	Sediments: 1200 mg/kg	[24]
Cuba/Havana City	Soil: 101 ± 51 mg/kg	[25]
Uganda/Kilembe copper mine and tailing sites	Tailings: 10,217 mg/kg Sediments: 4110 mg/kg Water: 1.9–61 μg/L	[26]
India/Ghaziabad	Soil: 122 mg/kg	[27]
Brazil/Carajas-Amazon	Water: 50–60 nmol/L	[28]
China/Dexing copper mine sewage station	38.24–47.86 mg/L	[29]
China/Liaodong Bay	Water: 6.8–11.9 μg/L	[30]

**Table 2:** Summary of copper removal efficiency based on different treatments.

Techniques	Materials/Reactors	Removal Efficiency of Cu	References
Membrane separation	Hydrophilic polyurethane modified cellulose acetate ultrafiltration membranes	92%	[140]
	Cellulose acetate based biopolymeric mixed matrix membranes	84–88%	[141]
	Chitosan-cellulose acetate-TiO_2_ based membrane	97%	[142]
Ion exchange	Y zeolite ion exchangers	64%	[143]
	Ion exchange resin	99.14%	[144]
Electrochemical reaction	Bipolar disc reactor	90.1%	[145]
	Continuous electrochemical cell	91%	[146]
	Bioelectrochemical and electrochemical systems	99.9%	[147]
Chemical precipitation	OM in waste distillery slops—precipitation/coagulation	92%	[148]
	Synthetic nesquehonite	99.97%	[149]
	struvite	99.9%	[150]
Adsorption	Hexagonal boron nitride	92%	[151]
	Zeolite, bentonite, and steel slag	98.47–99.98%	[152]
	Agro-industrial waste	89%	[153]
Biotechnology	*Stenotrophomonas maltophilia*	88%	[154]
	Microalgae	>95%	[155]
	*Aspergillus australensis* Biomass	79%	[156]

**Table 3:** Summary of different copper ion removal technologies [3,157,158].

Technology	Advantages	Disadvantages	Application Scenarios	Cost
Membrane filtration	Excellent performance in scale-up applications, such as excellent heavy metal removal, high efficiency, ease of operation, and low space requirements	Membrane fouling, capital cost, maintenance and operational cost, less efficient in case of lower metal ion concentration	Suitable for both high- and low-concentration copper-polluted water; selection of the right polymer/micellar agent is required to improve the rejection efficiency	Treatment cost of membrane fouling
Reverse osmosis	Effective removal of metals from wastewater	Membrane scaling problems, low water permeability, high RO operating pressure due to internal concentration polarization, low water flux, and high energy consumption	Use in drinking water	
Ion exchange	Selective removal of heavy metals, high treatment capacity, high metal removal rate	Fouling and maintenance costs, high capital cost of equipment and instruments, high operational as well as resin regeneration cost	Treatment of water bodies polluted by a specific metal element, not suitable for large-scale application	High cost of synthetic resin, pollutant recovery costs
Electrochemical reaction	Reduced chemical consumption, recovery of pure metals, effective removal of desired metals, suitable for initial high concentration contamination remediation	Low current effect and selectivity, high power consumption	Electrochemical methods, such as electrodialysis, electrocoagulation, electrodeposition, and capacitive deionization, are capable of removing Cu(II) by different mechanisms and are therefore suitable for a wide range of copper concentrations	Electricity costs
Chemical precipitation	Low metal concentration in the effluent achieved. This approach can be adapted to handle large quantities of wastewater. Simple to use	High chemical requirement, pH maintenance at optimum level, handling of colloidal particle sludge disposal problem. A large number of factors, such as temperature, pH, precipitant concentration, etc., have to be monitored when implementing this technique, which is quite difficult	For the treatment of concentrated copper wastewater, the preferred method is precipitation	Sludge disposal cost
Adsorption	Highly effective for removing heavy metals within permissible limits; the desorption process can produce a concentrated Cu(II) stream with recovery potential	Chemical regeneration requirement, fouling and corrosion of treatment plant, disposal of exhausted adsorbents, preparation of the adsorbent involve high costs, such as in the case of activated carbon, loss of adsorption capacity by the adsorbent in each cycle, frequent regeneration, which reduce the simplicity of the adsorption process	When treating diluted wastewater, adsorption is preferred due to its simplicity and effectiveness	Cost of desorption and regeneration

## Data Availability

Not applicable.
